# Atomic-Scale Defects and Edge Engineering of ZrSe_2_ Nanosheets: Correlated Microscopy, Spectroscopy and DFT Study with Implications for Quantum Device Applications

**DOI:** 10.1021/acsanm.5c03451

**Published:** 2025-10-21

**Authors:** Sharieh Jamalzadeh Kheirabadi, Luca Persichetti, Lida Ansari, Gabriele Anselmi, Paul K. Hurley, Luca Camilli, Farzan Gity

**Affiliations:** † MicroNano Systems, 261183 Tyndall National Institute, University College Cork, Cork T12 R5CP, Ireland; ‡ Department of Physics, 9318 University of Rome Tor Vergata, via della Ricerca Scientifica 1, Rome 00133, Italy; § School of Chemistry, University College Cork, Cork T12 CY82, Ireland

**Keywords:** 2D-transition metal dichalcogenides (TMDs), scanning tunnelling microscopy/spectroscopy (STM/STS), density functional theory (DFT), ZrSe_2_, point defect, grain boundary, edge passivation

## Abstract

We present a comprehensive study of the atomic-scale electronic behavior of ZrSe_2_, focusing on the effects of intrinsic point defects, grain boundaries, and edge configurations. Using a combination of low-temperature scanning tunnelling microscopy/spectroscopy (STM/STS) and density functional theory (DFT), we identify and characterize the spectroscopic fingerprints of various intrinsic point defects, including vacancies, antisites, and interstitials, and reveal how these features perturb the band edges or introduce in-gap states. These defect-induced features are shown to significantly influence the local electronic properties of ZrSe_2_. Our analysis of grain boundaries identifies shear-type interfaces that shift the Fermi level without introducing deep in-gap states, thereby preserving the semiconducting character of pristine ZrSe_2_. In contrast, the edge configuration has a pronounced effect on the electronic structure, with armchair and zigzag edges exhibiting distinctly different behaviors. While the former is characterized by a prominent peak near the valence band edge, indicating the presence of edge-localized states and a clean semiconducting character, the latter instead introduces a significant density of states at midgap and within the upper half of the bandgap. These findings offer atomic-level insights into the interplay between defects, edge chemistry, and electronic behavior in ZrSe_2_, establishing a framework for defect- and edge-state engineering in two-dimensional semiconductors for nanoelectronics and quantum device applications.

## Graphical abstract

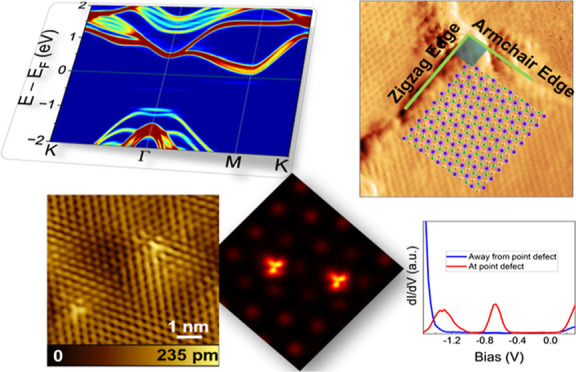

## 1 Introduction

Transition metal dichalcogenides (TMDs) constitute a broad family of layered materials with the general formula MX_2_, where M represents a transition metal from group IV (Ti, Zr, Hf), group V (V, Nb, Ta), or group VI (Mo, W), and X is a chalcogen atom (S, Se, Te). The atomic layers of TMDs are loosely bond together by van der Waals (vdW) forces, making it easy to separate them into single or few-layered nanosheets. Over the past decade, TMDs have gained significant attention due to their exceptional stability,^1^ large current density,^2,3^ high carrier mobility,^4^ tunable bandgaps,^5^ and outstanding mechanical and optical properties.^6,7^ These attributes have fuelled interest in their potential applications in electronic devices^3,4,8^ and photocatalysis.^9^

ZrSe_2_ is among the first two-dimensional materials to exhibit properties comparable to those of silicon in a technological context. The bandgap of ZrSe_2_ remains relatively unchanged as the material transitions from bulk to single-layer form, staying indirect and ranging from 0.9 to 1.2 eV.^10^ This stability suggests that ZrSe_2_ can withstand the high variability encountered in system-level applications. Moreover, the bandgap size is similar to that of silicon, being small enough to support low-voltage operation in future electronics, yet large enough to enable current on/off ratios greater than 10^6^. Lastly, ZrSe_2_ benefits from a native oxide (ZrO_2_), which resembles silicon’s oxide but offers the added advantage of technologically desirable high-k insulators.^11^

Nanoribbons are considered more promising than layered and bulk materials for realizing next-generation electronic devices due to their tunable electronic properties.^12–16^ For instance, graphene nanoribbons (GNRs) can exhibit either metallic or semiconducting behavior, depending on the direction in which they are cut.^13^ MoS_2_ nanoribbons display ferromagnetic and metallic characteristics at the zigzag edge, while they are nonmagnetic and semiconducting at the armchair edge.^17^ Zigzag MoS_2_ nanoribbons are also promising candidates for use as cathode materials in Li-ion batteries, offering high power densities and fast charge/discharge rates.^18^

On the other hand, the presence of some zero-dimensional (0D) defects on or beneath the surface of a TMD film is largely unavoidable, arising from the environment, the substrate, and the synthesis conditions. These point defects can include interstitial impurities, vacancies, self-interstitial impurities, substitutional impurities, or a combination of these. They can create surface and/or midgap electronic states, which profoundly alter the material’s intrinsic electronic properties and may be either beneficial or undesirable. Recent studies have explored the presence and effects of point defects in TMDs,^19–27^ providing insights into their impact on the electronic properties of the materials and helping assess their significance and potential applications.

ZrSe_2_ bulk crystal has been synthesized from elemental precursors through chemical vapor transport (CVT), with iodine used as a transport agent.^11,28^ Previous studies on synthetic diselenide crystals have identified them as n-type semiconductors, with optical absorption,^28–30^ angle-resolved photoemission spectroscopy (ARPES),^31^ and scanning tunnelling spectroscopy (STS)^20,32–34^ measurements confirming that bulk ZrSe_2_ has an indirect bandgap of 1.2 eV. More importantly, recent scanning tunnelling microscopy (STM) studies have found that 0D defects in ZrSe_2_ locally induce charge density wave (CDW) reconstruction which is not observed in pristine (i.e., nondefective) areas. In particular, the presence of these defects results in a local n-doping of the crystal which shifts the conduction band minimum (CBM) below the Fermi level, leading to a semiconductor-to-metal transition and the formation of a commensurate and nondispersive CDW reconstruction with a periodicity length of 2a (where a is the lattice parameter of ZrSe_2_).^35,36^ This finding underscores the importance of defects in defining the properties of TMDs.

In this research, we conducted a correlated combined simulation and experimental study to investigate the effects of various point and line defects in ZrSe_2_. To examine the defect’s properties, we employed low-temperature scanning tunnelling microscopy and spectroscopy (STM/STS), supported by density functional theory (DFT) calculations. Scheme 1 shows the modeled atomic structure of the ZrSe_2_ nanosheet, along with a schematic representation of the STM measurement setup which provides an overview of the atomic lattice and experimental configuration, laying the foundation for understanding the structural and electronic characteristics of the observed defects. Our findings provide a new knowledge and comprehensive understanding of the local impact of the line and point defects on the electronic properties of ZrSe_2_ and hence on their potential applications.

**Scheme 1:**
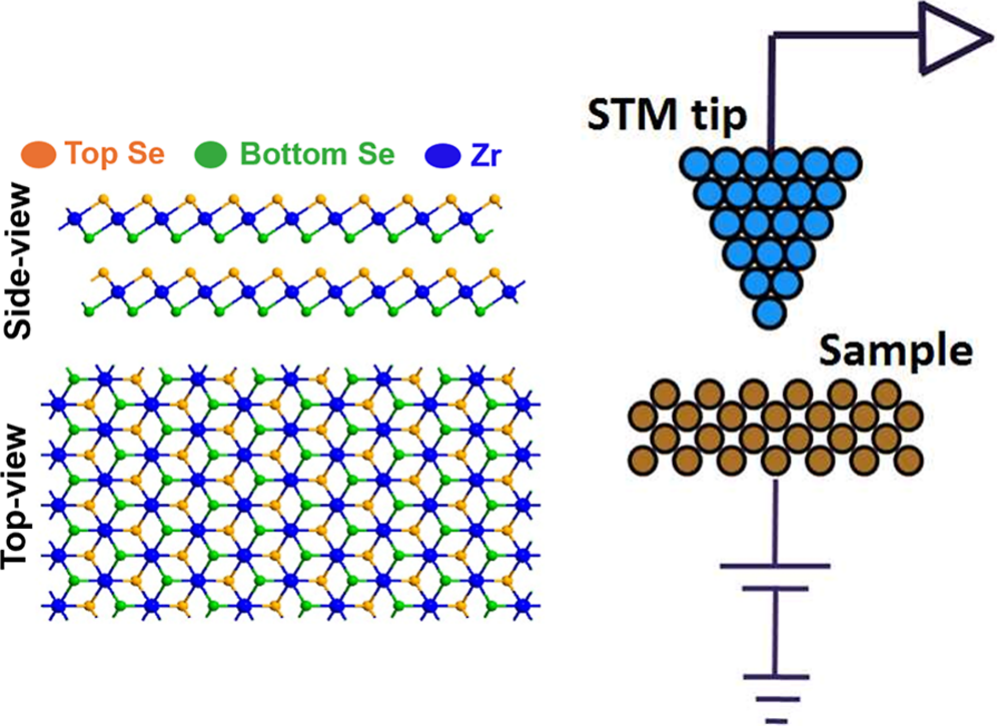
Modelled Atomic Structure of the ZrSe_2_ Nanosheet along with the Corresponding Schematic of the STM Measurements

## 2 Results and Discussions

### 2.1 Defective ZrSe_2_–Point Defects

Figure 1a,b show STM topography images of a pair of point defects, which were frequently observed on the ZrSe_2_ surface in our measurements, obtained for filled (negative bias potential) and empty states (positive bias potential), respectively. At negative bias (Figure 1a), the defects appear as two bright-contrast triangular features, each consisting of three lobes radiating from a central site at ∼120° intervals, exhibiting 3-fold (C_3_) rotational symmetry. From Figure 1b, it can be noticed that, in the same area imaged at positive bias, the three-lobe structure of the defects is no longer visible. Away from the defects, at both bias polarities, the surface structure corresponds to that expected for 1T ZrSe_2_,^37,38^ with the STM revealing the hexagonal symmetry of the top Se layer of the ZrSe_2_ crystal. Interestingly, STS dI/dV spectra acquired on the point defect (at the three-lobe site labeled as 2 in the inset of Figure 1c), exhibit distinct features compared to spectra taken away from the defects, such as at location 1 in Figure 1c. At the latter site, we observe a bandgap consistent with the expected value of 1.2 eV,^35,36^ whereas at the point defect site two prominent defect-induced electronic states appear, one near the center of the bandgap and the other close to the valence band edge.

**Figure 1:**
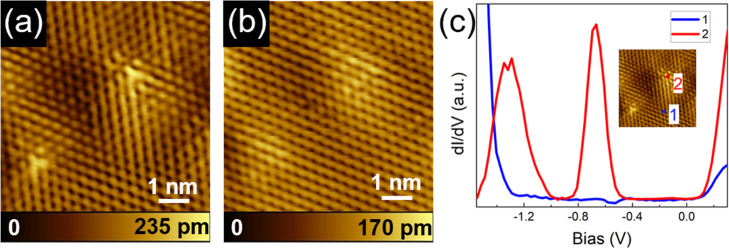
Dominant point defect type observed experimentally in freshly exfoliated ZrSe_2_ crystal. STM topography image of defect sites on ZrSe_2_ surface: (a) *I* = 120 pA, *V* = −500 mV, and (b) *I* = 120 pA, *V* = +100 mV. (c) Experimental STS spectra (acquired at 10 K with feedback disabled at a set point of *I* = 120 pA, *V* = −500 mV) taken outside the point defect (blue curve, location 1) and on the defect (red curve, location 2). The exact acquisition locations are indicated in the inset.

To unveil the nature of these point defects, we have systematically analyzed five distinct types of point defects in ZrSe_2_ using first-principles calculations. This theoretical approach enables a detailed understanding of how these point defects alter the local electronic properties and contribute to changes in conductivity and potential device performance. These include a zirconium vacancy (Zr-vac), a selenium vacancy (Se-vac), a zirconium antisite defect, where a Zr atom occupies a Se atomic site (Zr-antisite), a selenium antisite defect, where a Se atom occupies a Zr site (Se-antisite), and Zr interstitial (Zr-int). To model the point defects, we employed a 5 × 5 × 3 supercell of pristine 1T-ZrSe_2_, which sets the minimum periodic defect–defect separation to ∼1.83 nm.

Figure 2 presents the relaxed atomic structures and simulated STM images based on fully relaxed atomic structures predicted by DFT for each of the aforementioned defect types. These simulated DFT-STM images serve as a valuable reference for interpreting the features observed in the experimental STM data, enabling us to infer the likely nature of the defects present in ZrSe_2_. To facilitate a more accurate comparison with the experimental results, the side- and top-views of the relaxed atomic configurations of the defective ZrSe_2_ are presented in the top row of Figure 2, while the DFT-STM images are displayed in the middle row.

**Figure 2:**
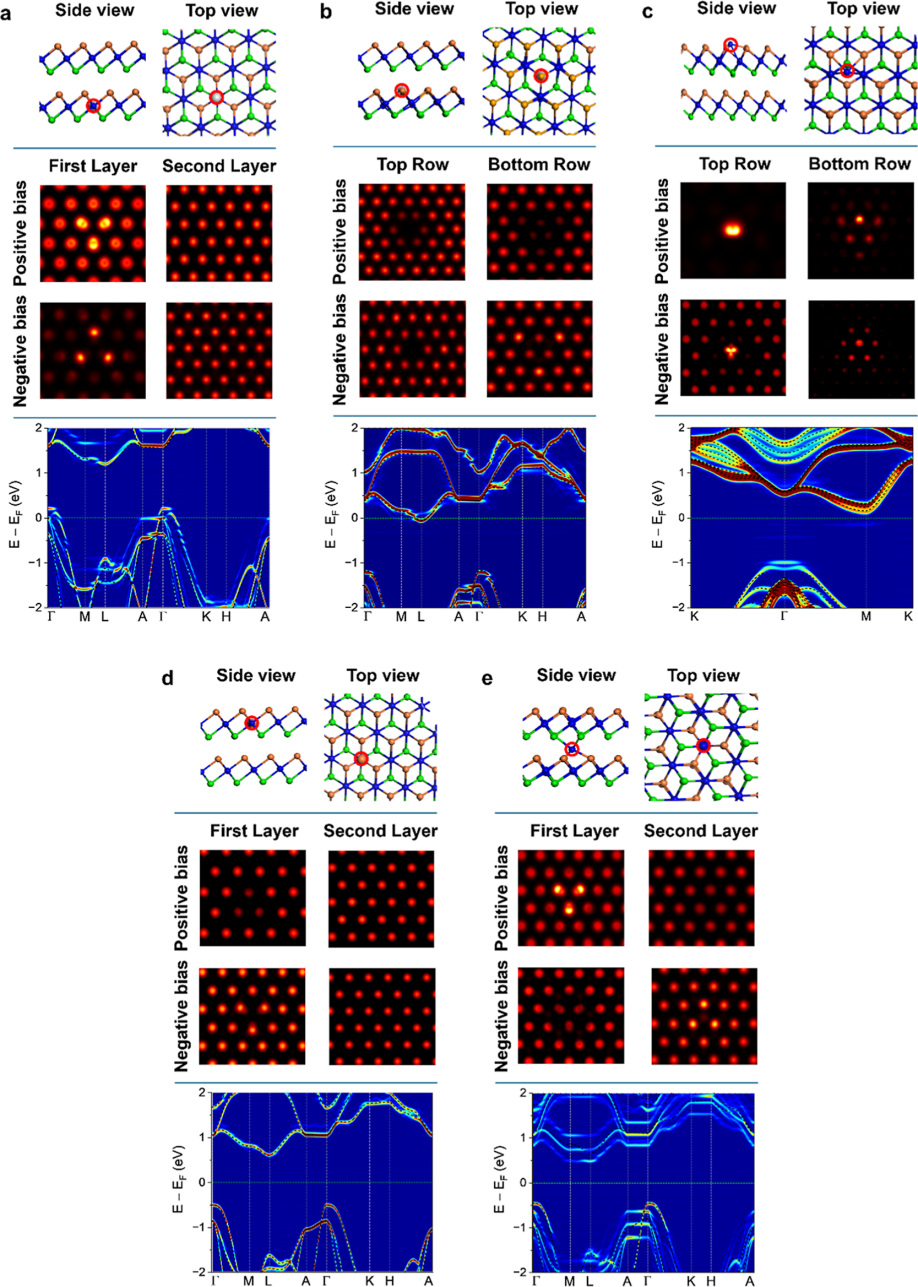
DFT analysis of various point defects in ZrSe_2_. Side and top views of the relaxed atomic structures, DFT-STM images, and unfolded band structures of (a) Zr-vac, (b) Se-vac, (c) Zr antisite, (d) Se antisite, and (e) Zr-int, respectively. In the unfolded band structures, red and blue indicate the two ends of the spectral weight spectrum, respectively. Darker shades correspond to minimal deviation from the pristine band structure, indicating strong overlap with the intrinsic (defect-free) structure, shown by black dashed line. Note that the Zr antisite is modeled in a 4L structure, which enables an antisite configuration at the top surface. Red circles mark the locations of the point defects.

To elucidate the impact of point defects on the electronic band structure of ZrSe_2_ thin films and bulk configurations, the unfolded band structures (see the Methods section) corresponding to various point defect types introduced at a concentration of ∼0.3% are presented as contour plots in the bottom row of Figure 2. The electronic states associated with the presence of defects are largely localized and, due to the broken translational symmetry, lack well-defined crystal momentum. However, by projecting these defect-perturbed states onto the eigenstates of the pristine system (which do possess well-defined wave vectors), it is possible to assign a dominant or distributed momentum character to each localized state. The intensity of this projection is depicted via contour shading. The horizontal axis represents the crystal momentum (wave vector) in the Brillouin zone, while the vertical axis shows the energy of the electronic states, referenced to the Fermi level (*E*_F_). For comparison, the band structure of pristine ZrSe_2_ is superimposed as dashed black lines, aligned with the conduction band minimum of the defective system to facilitate a consistent reference point.

Interestingly, isolated Zr-vac (Figure 2a) and Se-vac (Figure 2b) in ZrSe_2_ do not introduce deep defect states within the bandgap, in contrast to similar point defects in other TMDs such as MoS_2_, PtSe_2_, WS_2_, and PtTe_2_, where such vacancies lead to pronounced in-gap states.^33,37–39^ The influence of each defect on the electronic structure is evident when compared to pristine ZrSe_2_. In particular, the Zr-vac induces perturbations that are most prominent near the valence band edge, whereas in the case of the Se-vac, the band perturbation emerges near the conduction band edge. Moreover, based on the position of the Fermi level, the Zr-vac results in strongly p-type behavior, while the Se-vac leads to n-type characteristics.

The defect states of the Zr-antisite structure (Figure 2c) are situated in the bandgap, and the Fermi level is shifted toward the conduction band. Conversely, the Fermi level lies near the center of the bandgap for both the Se antisite and Zr-int defects (Figure 2d,e), suggesting a more intrinsic or compensated electronic character. Notably, the Se antisite shows no significant band perturbation, whereas the Zr-int defect induces a significant perturbation. The corresponding PDoS for the different point defects are presented in Figure S1 of the Supporting Information. These observations highlight the distinct electronic signatures introduced by different defect types in ZrSe_2_.

By comparing the experimental STM image in Figure 1 with the simulated images in Figure 2, we note that the Zr antisite (Figure 2c) shows a 3-fold (C_3_) rotational symmetry with three lobes separated by 120° at negative bias, in agreement with the experiment. At positive bias, however, this C_3_ symmetry is lost, again consistent with the experimental observation. This behavior is a peculiarity not seen in other defect types. For instance, in the case of Zr-vac (Figure 2a), triangular features appear in the simulations at both bias polarities, but rotated by 180° depending on the bias sign. The structural resemblance therefore provides an initial indication that the defect observed in Figure 1 is a Zr antisite. However, because structural resemblance alone may not be sufficient to confirm this identification, we also extend our comparison to spectroscopic signatures. Specifically, we investigate the characteristics of double Zr antisite point defects located adjacent to each other, as shown in Figure 3a. The DFT-obtained STM image (DFT-STM), shown in Figure 3b, again demonstrates a close agreement with experiment. To further assess this identification, we examine the simulated PDoS of the double Zr antisite (Figure 3c). Two distinct sets of defect-induced electronic states are observed: one near the center of the bandgap and another close to the valence band edge. The position of the defect states inside the bandgap closely matches the experimental STS spectrum acquired on the defect (red curve in Figure 1c). Importantly, the presence of defect states within the bandgap is unique to the Zr antisite among the five different detect types investigated (see Figure 2), thereby providing strong evidence that the experimentally observed defect in Figure 1 is indeed a Zr antisite. It is noted that while the bandgap value and the relative positions of the defect states with respect to the band edges resemble the experiment, the absolute energy scale of the theoretical PDoS is rigidly shifted relative to the experimental STS. Such quantitative discrepancies, which also appears for line defects discussed below, is likely due to factors such as tip-induced gating and band bending effects, and work function mismatch between the STM tip and the sample, and surface relaxations or unintentional defects that are not accounted for in the calculations.

**Figure 3:**
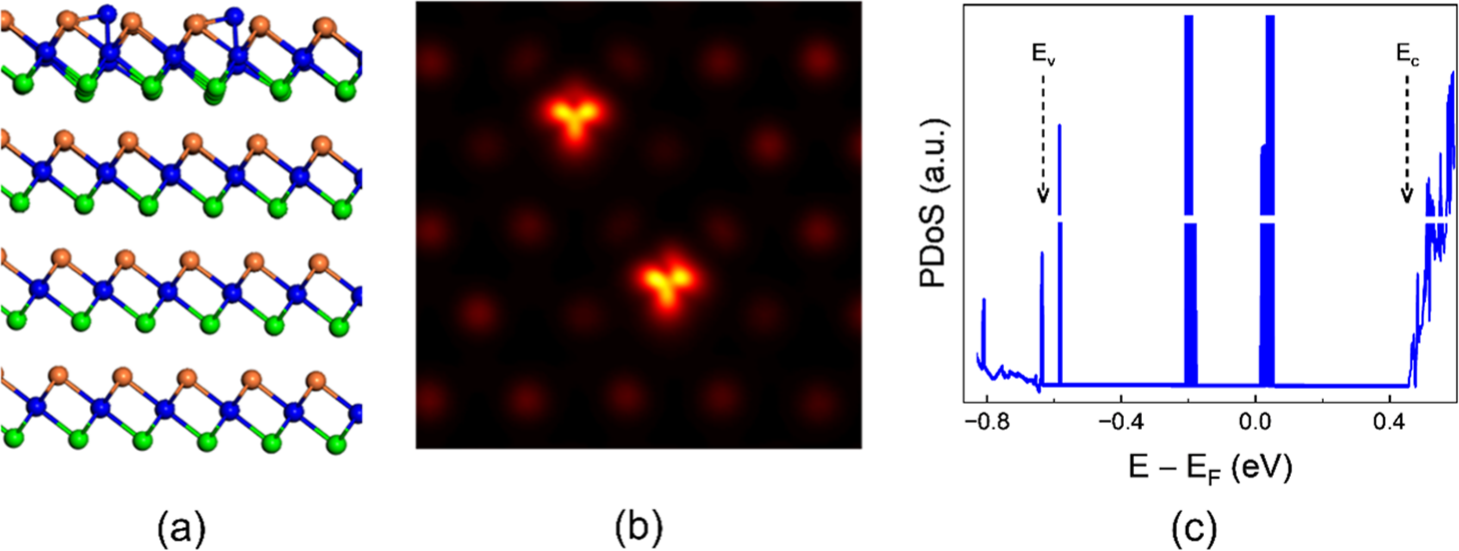
Double-Zr antisite point defect in ZrSe_2_. (a) Atomic structure, (b) DFT-STM image calculated at negative bias, and (c) theoretical PDoS of the pair of Zr antisite point defect. The band edges are indicated by the *E*_v_ and *E*_c_ labels and arrows.

### 2.2 Defective ZrSe_2_–Line Defects

Grain boundaries (GBs) in two-dimensional materials have been reported to form through several mechanisms, including the coalescence of growing flakes during synthesis, the accommodation of nonstoichiometry within the sample, or as a result of postgrowth processes such as electron irradiation or thermal annealing.^40^ These boundaries emerge from the atomic stitching of adjacent domains that possess different crystallographic orientations. Since these domains nucleate randomly across the substrate, the resulting GBs can exhibit a variety of orientation angles.^41^ Based on the relative orientation between adjoining domains, grain boundaries are typically categorized into tilt GBs, twin GBs, and mirror twin boundaries.

GBs in TMD crystals are often present and are thought to be the main cause of the poorer performance of electrical devices when compared to the theoretical predictions; yet experimental observations of GBs are quite rare. Our STM measurements reveal the presence of a specific type of GB in ZrSe_2_, commonly referred to as a “shear GB”. Figure 4a shows a topographic STM image of a shear GB we have observed in ZrSe_2_ with the measured height of around 0.031 nm (Figure 4b)—a much smaller height than the expected monatomic step height of 0.616 nm.^42^ In the shear GB, one grain is laterally displaced relative to the other, with no rotational misalignment. As illustrated in Figure 4c, the displacement observed in our STM images corresponds to half the length of a Zr–Se bond. A ball-and-stick model of the shear GB is displayed in Figure 4d. The black dotted lines in panels (c,d) show the interface between the two grains of this GB, while the black dashed lines indicate the displacement. We have also investigated the electronic properties of this type of GB in ZrSe_2._ To avoid spurious effects from edge states and to capture the localized GB-induced states, the simulated GB structure was constructed with a length of 26.19 nm and a vacuum spacing of 2 nm between periodic images. We note that the unsaturated bonds at the GB defect were not passivated. As shown in Figure 4e, the STS data collected on the GB exhibit no in-gap states, in strong agreement with our theoretical predictions obtained from DFT calculations (Figure 4f). Additionally, the Fermi level is observed to shift closer to the CBM, suggesting a potential n-type character induced by this defect. The PDoS of atoms neighboring the shear GB is provided in the Supporting Information (Figure S2).

**Figure 4:**
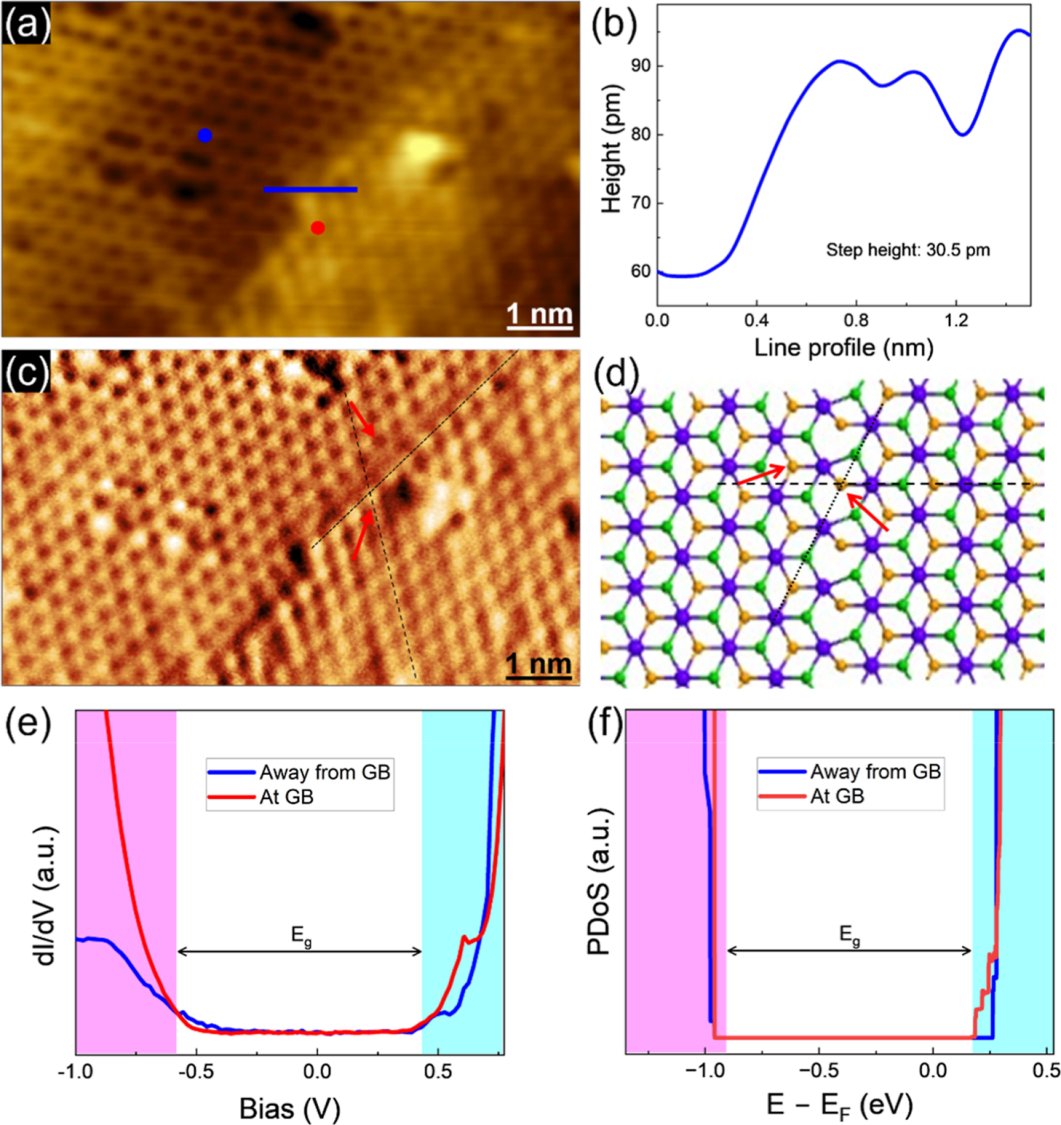
Shear grain boundary (GB) in ZrSe_2_. (a) Topographic STM image of a shear GB (*I* = 100 pA, *V* = −1 V) (left), and (b) the line profile collected across it. (c) STM current image acquired along with the topographic image in (a). It is shown to better observe the atomic arrangement close to the GB. (d) Atomic structure after relaxation of the shear GB defect. The black dotted and dashed lines indicate the GB and a displacement, respectively. The displacement is equal to half the Zr–Se bond length. The red arrows point to the Se atoms at the two sides of the GB. (e) Experimental STS (feedback was disabled at a set point of *I* = 120 pA, *V* = −1 V; acquired at 10 K), and (f) projected density of states (PDoS) of the GB and away from it. In panel (e), the red and blue spectra correspond to the locations marked by the red and blue dots in the STM image in panel (a). The shaded regions in the STS and PDoS plots highlight the valence and conduction bands.

Furthermore, we have also investigated how different edge terminations influence the electronic structure of ZrSe_2_. Specifically, we examine two distinct edge configurations: zigzag-terminated edges, characterized by a single atom at the edge, and armchair-terminated edges, which feature three atoms at the edge. Figure 5a presents a topographic STM image of these edges. Experimentally, a step height of 0.616 nm (Figure 5b) is measured, which is in agreement with the interlayer distance of ZrSe_2_ reported in the literature.^42^ For the DFT simulations, nanoribbon structures with a width of 6 nm were considered for both zigzag and armchair configurations. The relaxed atomic structures show good agreement with the STM image, where both edges are oriented perpendicular to each other, forming an angle of 90° (Figure 5c,d).

**Figure 5:**
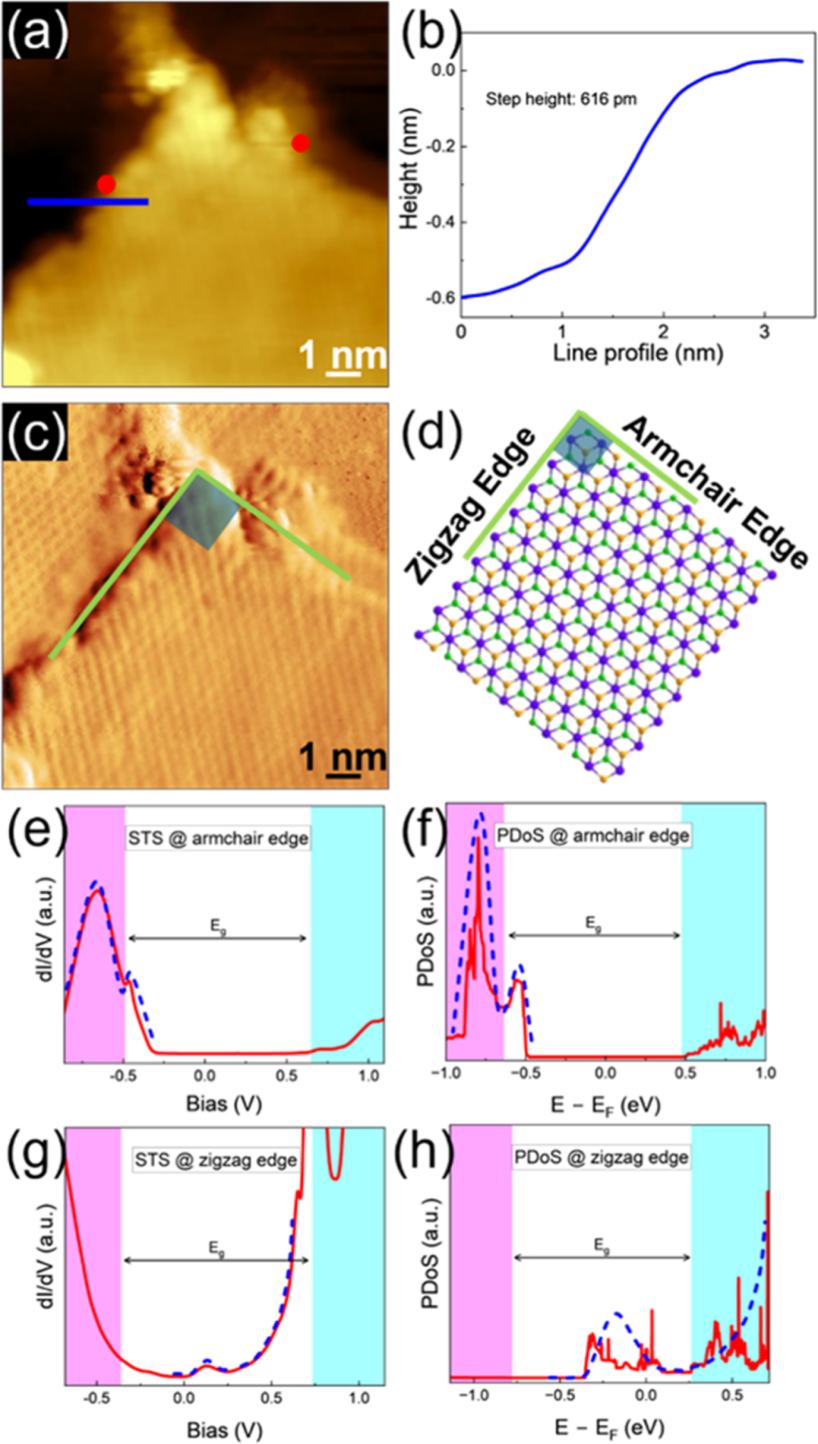
Armchair- and zigzag-terminated edges in ZrSe_2_. (a) Topographic STM image (*V* = −1.1 V, *I* = 200 pA) and (b) the line profile taken across the step edge highlighting the monatomic step height. (c) STM current image collected with the same parameters of (a). (d) Relaxed atomistic model of the armchair and zigzag edge terminations in ZrSe_2_. (e) STS spectrum (feedback disabled with set point: *I* = 2 nA, *V* = −1.5 V) and (f) PDoS calculations for the armchair edge configuration. (g) STS spectrum (feedback was disabled at a set point of *I* = 2 nA, *V* = −1.5 V; acquired at 10 K) and (h) PDoS calculations for the zigzag edge configuration. The shaded regions in the STS and PDoS plots highlight the valence and conduction bands. The experimental STS were acquired on the locations marked by dots in panel (a). The color of the dot is the same as that of the corresponding STS curve. The blue dashed curves serve as guides to the eye, tracing the trend of defect-induced states and illustrating the strong agreement between the features observed in the experimental STS data and found in the simulated PDoS curves.

The STS acquired on armchair- and zigzag-terminated edges are presented in Figure 5e,g, respectively, while the calculated DoS for armchair- and zigzag-terminated bulk ZrSe_2_ configurations are reported in Figure 5f,h. The corresponding band structures are provided in Figure S3a,b in the Supporting Information. These results reveal a distinct contrast in the electronic behavior between the two edge types. The blue dashed curves in these figures trace the overall trend of changes in the electronic structure and show a consistent agreement between the experimental STS and simulated PDoS spectra.

Notably, for the armchair termination both the experimental and theoretical data reveal a prominent peak near the valence band edge, indicating the presence of edge-localized states. The absence of significant states within the bandgap region in both data sets suggests a clean semiconducting character, with no midgap states introduced by the edge termination. Additionally, the Fermi level lies closer to the midgap, suggesting an intrinsic semiconducting characteristic of the armchair-terminated ZrSe_2_ nanoribbons.

In contrast, the zigzag-terminated configuration introduces a significant density of states at midgap and within the upper half of the bandgap, as evident in both the STS and PDoS results. This behavior is markedly different from that of the armchair-terminated edge, where the bandgap remains largely preserved and the dominant features lie near the valence band edge. The presence of in-gap states near the conduction band in the zigzag case suggests that this termination substantially alters the local electronic environment. These modifications are likely rooted in differences in atomic coordination and bonding at the edge. Together, these findings highlight the critical influence of edge geometry on the electronic structure of ZrSe_2_ and emphasize the potential for edge engineering in tuning the material’s electronic and functional properties.

Furthermore, the structural characteristics of the step edges—specifically the bilayer-trilayer-bilayer (BL-3L-BL) configurations, which correspond to a monolayer step height consistent with experimental observations and featuring both zigzag and armchair terminations—have been examined. Detailed illustrations and analyses of these step edge structures are provided in Figures S4 and S5 of the Supporting Information, offering additional insight into their geometric and electronic properties. Furthermore, we explored the modulation of edge states through chemical functionalization in both zigzag- and armchair-edged nanoribbons, considering a range of edge terminations including unpassivated, −H, −OH, and −F passivation. The corresponding results are presented in Figures S6 and S7 of the Supporting Information.

## 3 Conclusion

This study provides an atomically resolved understanding of how intrinsic point defects, grain boundaries, and chemically functionalized edge terminations influence the electronic properties of the layered semiconducting material, ZrSe_2_. Using a combined low-temperature STM/STS and DFT approach, we identify the spectroscopic signatures of vacancies, antisites, and interstitials, and show how they perturb the local band structure and modulate the Fermi level. Grain boundary analysis reveals that shear-type interfaces shift band alignment without introducing deep in-gap states, preserving the material’s semiconducting behavior. At the edge level, we demonstrate that zigzag and armchair terminations respond differently to various passivations. In particular, fluorine induces a sharp midgap state at armchair edges while suppressing edge states in zigzag ribbons, offering a tunable platform for quantum or resonant transport functionalities. Together, these findings highlight ZrSe_2_’s versatility as a 2D semiconductor, where defects and interfaces can be purposefully engineered to modulate its electronic behavior. This work establishes a blueprint for atomic-scale control in future nanoelectronics and quantum devices.

## 4 Methods

### 4.1 Density Functional Theory Calculations

Density functional theory (DFT) calculations were carried out using the generalized gradient approximation (GGA) to describe the exchange–correlation potential, specifically employing the implementation available in the QuantumATK simulation package.^43^ A medium-basis set linear combination of atomic orbitals (LCAO) approach was employed, utilizing GGA norm-conserving pseudopotentials from the PseudoDojo library.^44^ For Brillouin zone integration, a k-point grid was generated using the Monkhorst–Pack scheme,^45^ achieving a density of approximately 10 k-points per Å^–1^. An energy cutoff of 105 hartree was applied to the discretized real-space grid. van der Waals (vdW) interactions were included using the Grimme DFT-D3 dispersion correction method.^46^ The bandgap was calibrated using the meta-generalized gradient approximation (M-GGA), yielding a value of 1.18 eV, in excellent agreement with experiment.^35,36^ The simulated lattice constant was found to be 0.38 nm, also in close agreement with the experimental value.^37,38^ The use of localized basis functions in this computational framework enabled the modeling of larger supercells, which in turn allowed for a lower concentration of vacancies, improving the physical accuracy of defect-related studies. Furthermore, A vacuum layer exceeding 2 nm was introduced in the in-plane direction to prevent interactions between periodic images of the nanoribbons, effectively simulating bulk-like behavior.

### 4.2 Unfolded Band Structure Calculations

As the supercell size increases, the corresponding first Brillouin zone proportionally shrinks. This results in significant folding of the electronic bands into the reduced Brillouin zone, which can obscure the underlying dispersion relations present in the primitive cell. To facilitate a direct comparison between the supercell band structure and that of the primitive cell, a technique known as band unfolding is employed.^47–49^ Band unfolding maps the eigenstates of the supercell onto the Brillouin zone of the reference primitive cell, thereby recovering the Bloch character of the original states. When utilizing a linear combination of atomic orbital (LCAO) basis sets, especially in systems with perturbations such as point defects or impurities, this method also enables the identification of symmetry-breaking effects and their influence on the electronic structure.

### 4.3 STM/STS Experiments

All the STM/S experiments were performed using a commercial LT-STM Infinity system from Scienta Omicron operating at 10 K. A commercial ZrSe_2_ bulk crystal (from 2D Semiconductors) was exfoliated multiple times in air, then again inside the load lock chamber in vacuum, before being moved inside the analysis chamber (base pressure 5 × 10^–10^ mbar). Pt–Ir tips were obtained by mechanical cut of a Pt–Ir wire and used throughout the experiments. All the STM images were acquired in constant current mode. STM piezo calibration was performed on a reference Au(111) crystal at the same temperature of the experiment. After calibration, we obtain a lattice constant *a* = 0.37 ± 0.01 nm, consistent with the 1T crystal structure of the ZrSe_2_.^37,38^ The d*I*/d*V* spectra and maps were measured using a standard lock-in technique that applied an ac voltage with a peak value Δ*V* = 40 mV and a frequency *f* = 653 Hz. STM data were analyzed using the Gwyddion software.^50^

## Supplementary Material



## Notes

The SI presents a detailed investigation of the electronic structure of ZrSe_2_ through a series of computational and experimental plots. It begins with an analysis of the PDoS for ZrSe_2_ containing various point defects, followed by a localized PDoS study around a shear grain boundary line defect, alongside its atomic structure and band structure. The focus then shifts to edge effects, where band structures of armchair-edged and zigzag-edged nanoribbons are compared. To further explore step-edge features, STM images, atomic models, band structures, and PDoS are presented for both zigzag and armchair step-edge configurations. The role of chemical passivation is examined next, with relaxed atomic structures and spatially resolved PDoS plotted for zigzag-edged and armchair-edged nanoribbons under various terminations (–H, –OH, –F) (PDF)
