# Supplementary material for: Atomic-Scale Defects and Edge Engineering of ZrSe2 Nanosheets: Correlated Microscopy, Spectroscopy and DFT Study with Implications for Quantum Device Applications
Source: ACS Appl Nano Mater. 2025 Oct 21;8(43):20848–57. doi: 10.1021/acsanm.5c03451 (PMC12584101; doi:10.1021/acsanm.5c03451)
Supplement: Supplementary file 1 [file an5c03451_si_001.pdf]

Supporting Information

Atomic-Scale Defects and Edge Engineering of $ZrSe_2$ Nanosheets: Correlated Microscopy, Spectroscopy and DFT Study with Implications for Quantum Device Applications

Sharieh Jamalzadeh Kheirabadi[1,,¥], Luca Persichetti[2,¥], Lida Ansari[1], Gabriele Anselmi[2], Paul K. Hurley[1,3], Luca Camilli[2,*], and Farzan Gity[1,*]*

[1] MicroNano Systems, Tyndall National Institute, University College Cork, Cork, T12 R5CP, Ireland

[2] Department of Physics, University of Rome Tor Vergata, via della Ricerca Scientifica 1, 00133 Rome, Italy

[3] School of Chemistry, University College Cork, Cork, T12 CY82, Ireland

* co-corresponding authors:

sharieh.jamalzadeh@tyndall.ie,

luca.camilli@uniroma2.eu,

Farzan.Gity@tyndall.ie

¥ joint first-authors

Figure S1 displays the projected density of states (PDoS) for $ZrSe_2$ incorporating various types of point defects introduced in Figure 2 of the main text. The influence of each defect on the electronic structure is evident when compared to pristine $ZrSe_2$. In particular, for the Zr-vac, defect-induced states prominently appear in the lower half of the bandgap, while in the case of the Se-vac, these states emerge in the upper half of the bandgap. For the Zr-antisite defect, the defect states are also situated in the middle of the bandgap, and the Fermi level shifts into the conduction band. Conversely, for both the Se-antisite and Zr-int defects, the Fermi level is positioned near the centre of the bandgap, suggesting a more intrinsic or compensated electronic character. These observations highlight the distinct electronic signatures introduced by different defect types in $ZrSe_2$.

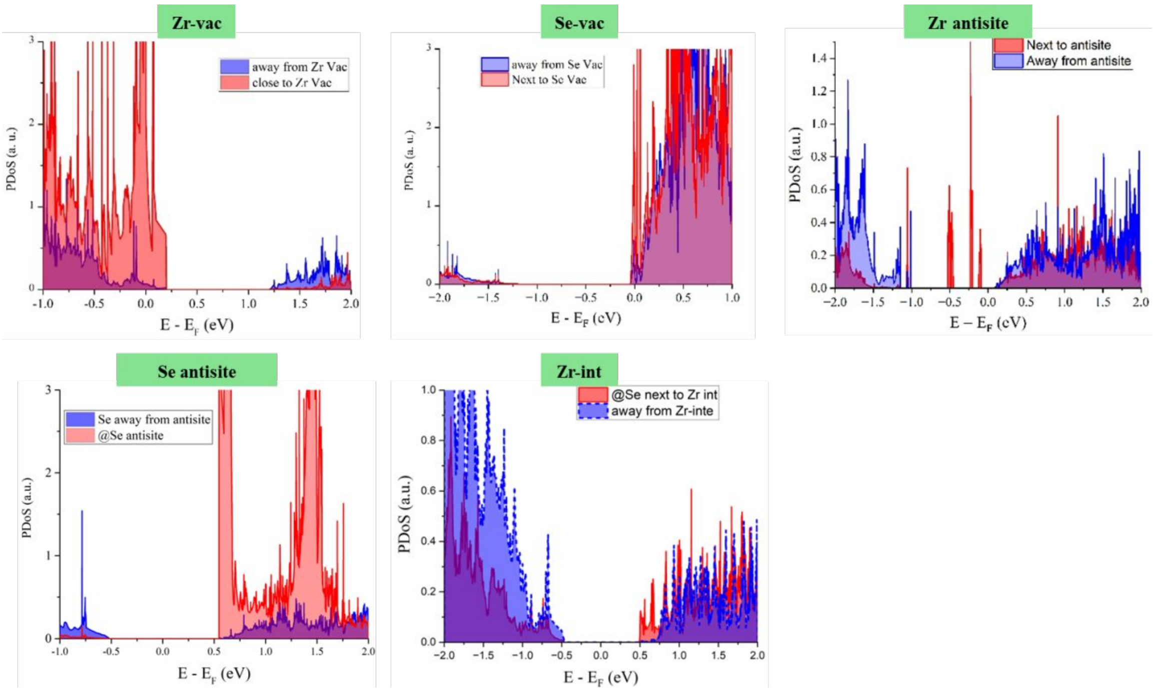

Figure S1: The PDoS plots of defective $ZrSe_2$ structures with various point defect types as Zr-Vac, Se-Vac, Zr antisites, Se antisites, and Zr interstitial.

To examine the influence of the surrounding atoms on the shear line defect, referred to as the grain boundary (GB), we analysed PDoS for each atom labelled in Figure S2(a). This atom-by-atom evaluation provides detailed insight into how the local electronic structure is perturbed in the vicinity of the defect.

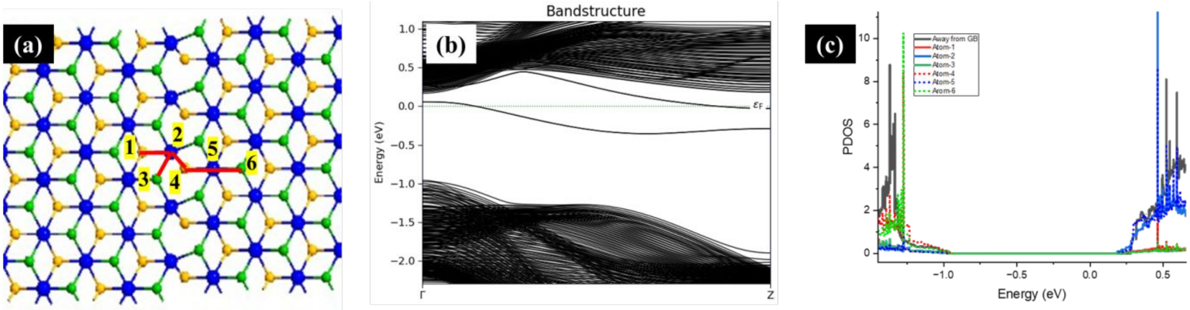

Figure S2: (a) Atomic structure of shear grain boundary line defect with the labelled surrounding atoms. (b) Band structure of the system. (c) PDoS at labelled atoms.

The band structures associated with armchair- and zigzag-terminated edges are presented in Figure S3, highlighting the distinct electronic properties that arise from different edge terminations.

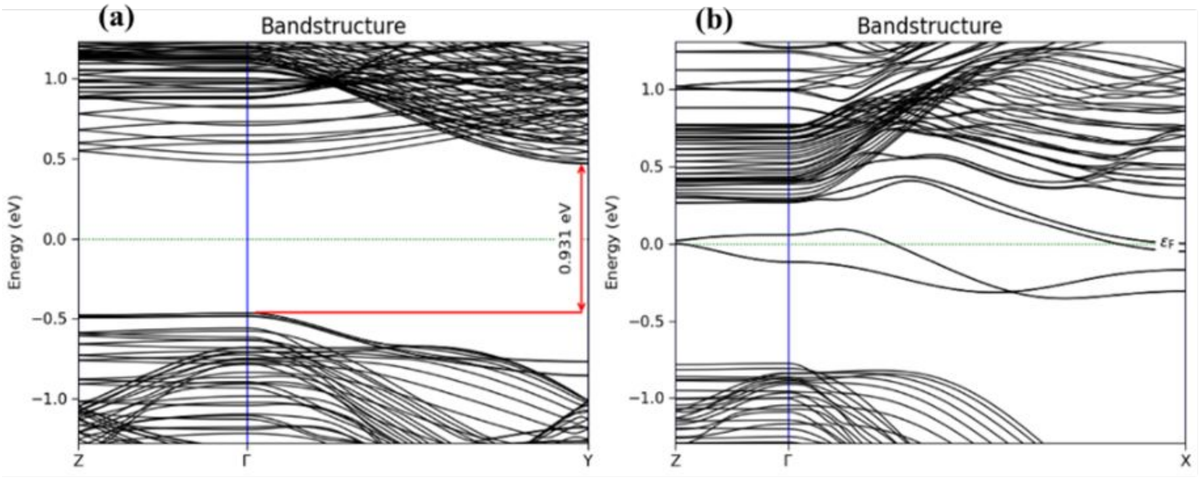

Figure S3: Band structures of (a) armchair-edged, (b) zigzag-edged $ZrSe_2$ nanoribbons.

Step edges represent a prominent structural characteristic in polycrystalline films, significantly influencing their overall physical and chemical properties. Gaining a deeper understanding of the electronic states associated with these features is crucial. It has been proposed that the energy landscape at step edges can vary qualitatively based on the strength of interlayer interactions. This consideration is particularly relevant for materials like $ZrSe_2$, which exhibit relatively strong interlayer coupling [S3]. From an application perspective, the potential to engineer self-contacting device architectures arises from the semimetal-to-semiconductor transition observed as the material's thickness is reduced. Such device geometries inherently involve the presence of step edges, making it essential to understand their electronic characteristics and the role they play in influencing charge transport and device functionality [S4]. In this study, we employ a combination of complementary experimental and theoretical techniques to explore and elucidate the electronic properties of step edges in $ZrSe_2$, aiming to provide comprehensive insights into their role in determining material behaviour and performance. Figure S4(a) presents a representative scanning tunnelling microscopy (STM) image of a monolayer (ML)-thick step-edge structure in $ZrSe_2$, characterized theoretically by a bilayer-trilayer–bilayer (BL-3L–BL) zigzag configuration. The corresponding atomic model of this structure is shown in Figure S4(b), where the bilayer (BL) region is partially overlaid by a third $ZrSe_2$ layer, forming a trilayer segment.

In terms of electronic properties, total band structure of the systems is shown in Figure S4(c) and the PDoS shown in Figure S4(d) reveals a bandgap of ~1 eV. Notably, the two distinct edge terminations at the zigzag BL-3L-BL interface introduce different localized edge states within the bandgap. These edge states are predominantly situated in the upper half of the bandgap, indicating their proximity to the conduction band and their potential influence on charge transport and surface electronic behaviour.

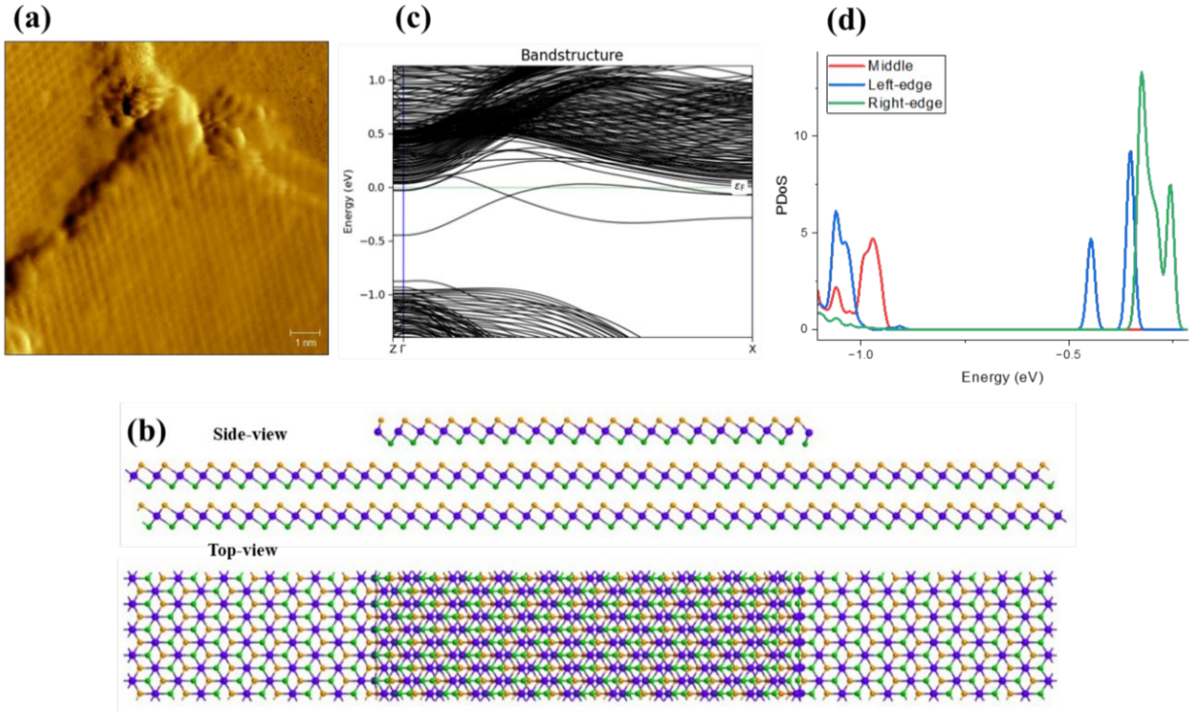

Figure S4: (a) STM current image of ML-thick step-edge structure in $ZrSe_2$ (zigzag edge). (b) Top- and side-view of the atomic model of this structure. (c) Band structure of the system. (d) PDoS plot of the pristine, i.e., middle of the structure, and the edges, showing drastic changes of the states at the edges.

A similar set of calculations have been performed for the BL-3L-BL armchair configuration, with the corresponding results presented in Figure S5. As clearly shown in the PDoS plot, edge passivation plays a critical role in modulating the electronic structure, particularly influencing the emergence and distribution of states near the valence band maximum (VBM). This indicates that the chemical nature of the edge terminations can substantially alter the electronic properties of the system, potentially impacting its overall stability and functionality.

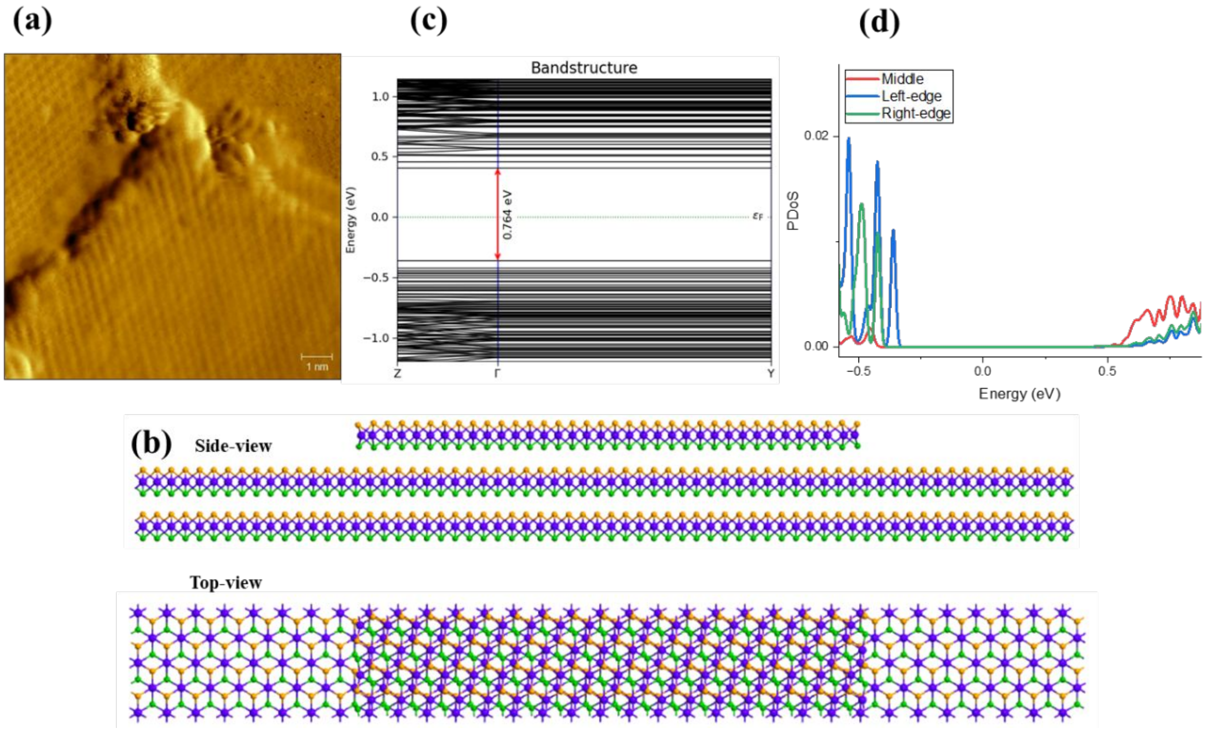

Figure S5: (a) STM current image of ML-thick step-edge structure in $ZrSe_2$ (armchair edge). (b) Top- and side-view of the atomic model of the structure. (c) Band structure of the system. (d) PDoS plot of the pristine, i.e., middle of the structure, and the edges showing the emergence of the states near VBM.

Modulation of edge states via chemical functionalization

As shown in Figure S6, the relaxed atomic structures and PDoS of 6 nm-wide zigzag-edged $ZrSe_2$ nanoribbons subjected to various edge terminations: unpassivated, –H, –OH, and –F passivated. All structures were fully relaxed, and the electronic properties were analyzed spatially across the ribbon - specifically at the left edge, center, and right edge.

In the unpassivated configuration, prominent edge-localized states appear predominantly in the upper half of the bandgap, indicative of unsaturated dangling bonds and electronic states associated with undercoordinated edge atoms. These defect-like states are absent in the ribbon's core, confirming their edge-specific origin.

–H passivation yields a similar distribution of states, with notable electronic density concentrated in the upper bandgap region. This partial passivation leads to a Fermi level shift towards the conduction band, imparting a distinctly n-type electronic character to the system. The persistence of edge states suggests incomplete electronic saturation, which may be advantageous for tuning carrier injection or contact resistance in device applications.

In contrast, –OH termination introduces continuous density of states across the Fermi level at the edges, effectively closing the bandgap and resulting in metallic edge behavior. This can be

attributed to the dual electron-donating and -accepting nature of hydroxyl groups, which enhance orbital hybridization and delocalization.

As evident in Figure S6, –F passivation in the zigzag configuration proves most effective, as mid-gap states are nearly fully suppressed, and the edge regions exhibit minimal electronic density near the Fermi level. This behavior reflects fluorine’s strong electronegativity and robust bonding, which effectively neutralize dangling bonds and stabilize the electronic structure.

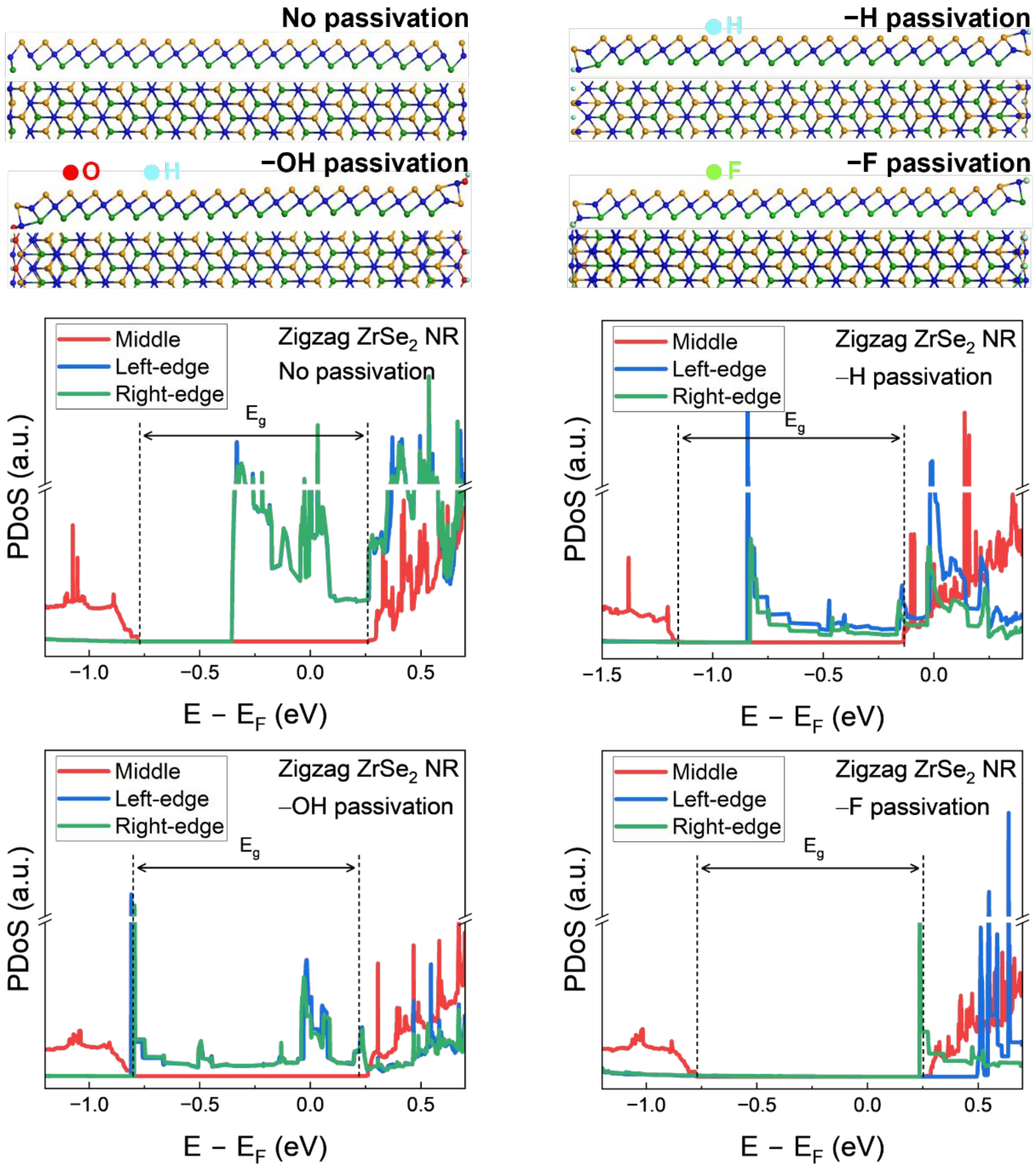

Figure S6: Electronic signatures of zigzag-edged $ZrSe_2$ nanoribbons. Relaxed top-view and side-view atomic structures (top) and spatially resolved PDoS plots (bottom) for the unpassivated nanoribbon and configurations terminated with –H, –OH, and –F groups. The PDoS is plotted separately for atoms located at the left edge, center, and right edge of each structure. Energy is referenced to the Fermi level.

As illustrated in Figure S7, the relaxed atomic structures and spatially resolved PDoS of 6 nm-wide armchair-edged $ZrSe_2$ nanoribbons under different edge functionalizations: unpassivated, –H, –OH, and –F terminations. The electronic properties were analyzed across three spatial regions: the left edge, center, and right edge of the ribbon.

In the unpassivated configuration, distinct edge-localized states appear near the valence band edge, on both the left and the right edges. These features suggest the presence of unsaturated dangling bonds at the armchair edge, although the mid-gap region remains relatively clean. The central region shows no significant electronic states near the Fermi level, confirming that the observed features are edge-induced.

–H passivation substantially reduces the density of edge states near the bandgap. A shift of the Fermi level towards the conduction band is observed, suggesting the emergence of an n-type character in the –H passivated edges.

Upon –OH termination, a high density of states emerges around the Fermi level at the edges, consistent with quasi-metallic edge behavior. This can be attributed to the polar nature and amphoteric chemistry of –OH groups, which enhance orbital hybridization and delocalization at the edge. Such features may be exploited in applications requiring conductive edge channels or tunable Schottky contacts.

Interestingly, as shown in Figure S7, –F functionalization leads to the formation of a strong, sharply localized mid-gap state at both edges. This contrasts with the zigzag case (Figure S6), where fluorine effectively suppresses mid-gap states. The emergence of a defect-like state at mid-gap in the armchair configuration suggests localized symmetry breaking or uneven orbital compensation due to strong electronegativity and possible overcompensation by fluorine. Such isolated mid-gap states can serve as active sites for resonant tunnelling or act as quantum emitters, including potential single-photon sources for quantum optics applications [S5].

Structural relaxation simulations reveal notable differences in Zr-Zr and Se-Se distances, as well as Zr-Se bond lengths and angles, for unpassivated and passivated $ZrSe_2$ edges with zigzag and armchair terminations. These results are presented in Table S1 of the SI.

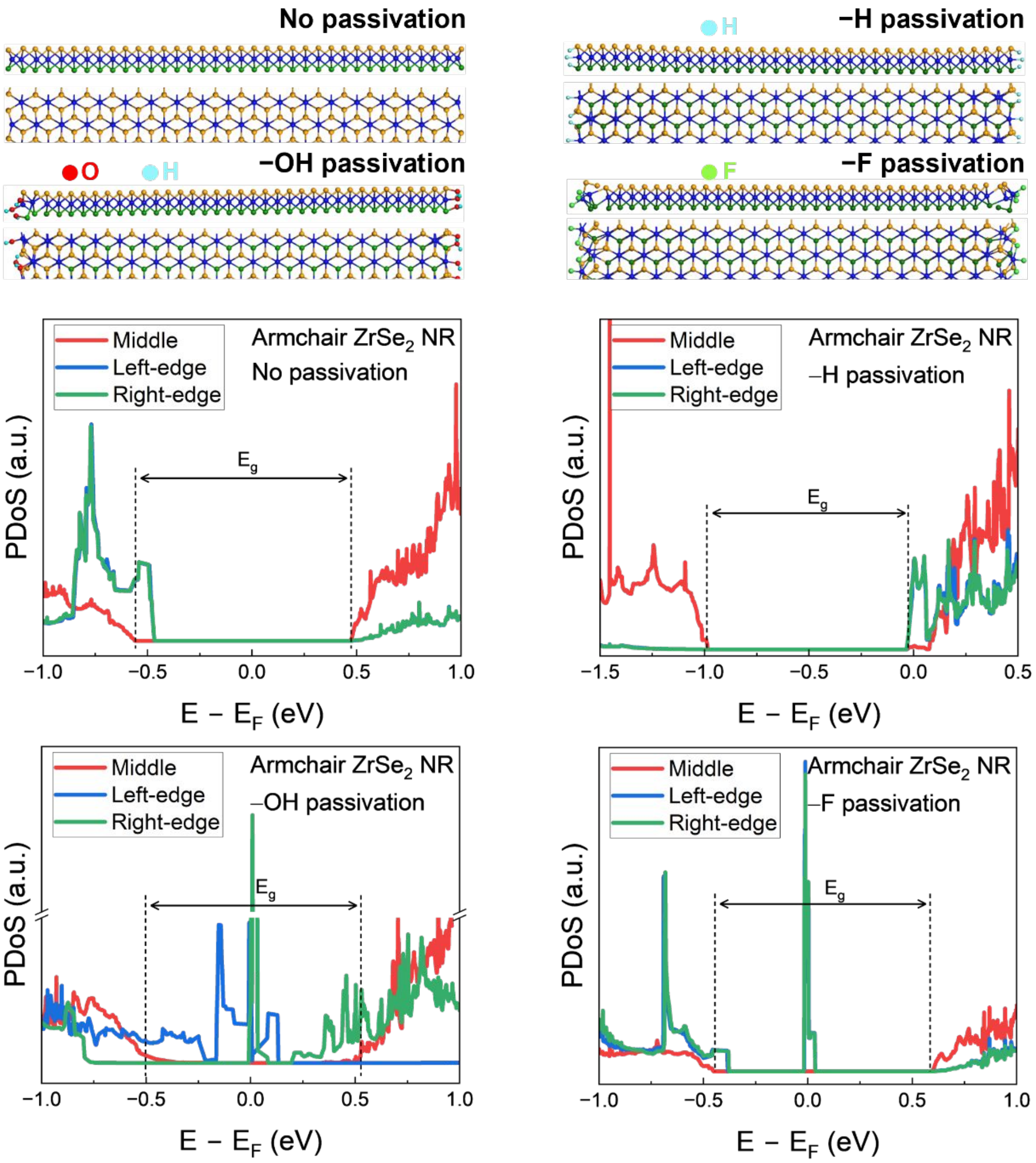

Figure S7: Electronic signatures of armchair-edged $ZrSe_2$ nanoribbons. Relaxed top-view and side-view atomic structures (top) and spatially resolved PDoS plots (bottom) for the unpassivated nanoribbon and configurations terminated with –H, –OH, and –F groups. The PDoS is plotted separately for atoms located at the left edge, center, and right edge of each structure. Energy is referenced to the Fermi level.

Table S1: Structural parameters of unpassivated and passivated $ZrSe_2$ nanoribbon edges with zigzag and armchair terminations, obtained from DFT relaxation.

Structure	Zr-Zr distance (A°)	Se-Se distance (A°)	Zr-Se Bond length (A°)	Bond angle (degree)
Zigzag-pristine	3.80	3.88	2.72	88.55, 91.37
Zigzag-unpassivated	3.31, 3.33	4.32, 4.36	2.53-2.86	71.38, 76.04
Zigzag-H	3.23, 3.25	3.73, 4.48	2.60-3.03	70.94, 73.47
Zigzag-OH	3.19, 3.35	3.67, 4.51	2.59-2.75	69.09, 75.97
Zigzag-F	3.21, 3.37	3.67, 4.47	2.58- 2.61	70.73, 76.35
Armchair-pristine	3.80	3.89	2.72	88.70, 91.40
Armchair-unpassivated	3.60, 3.95	3.73, 3.98	2.55, 2.65	86.29, 89.38
Armchair-H	3.75-3.78	3.97-4.02	2.74, 2.89	88.58, 96.27
Armchair-OH	3.70	3.73, 4.21	2.74-2.80	84.36, 98.27
Armchair-F	4.08, 4.74	3.73, 3.96	2.59-2.68	80.39, 107.56

References

[S1] Ørsted, A.; Scarfato, A.; Barreteau, C.; Giannini, E.; and Renner, C.; Doping Tunable CDW Phase Transition in Bulk 1T-$ZrSe_2$, *Nano Lett.* **2025**, 25, 4, 1729–1735, https://doi.org/10.1021/acs.nanolett.4c06377.

[S2] Ren, M. Q.; Han, S.; Fan, J. Q.; Wang, L.; Wang, P.; Ren, W.; Peng, K.; Li, S.; Wang, S-Z.; Zheng, F-W.; Zhang, P.; Li, F.; Ma, X.; Xue, Q-K.; and Song, C-L.; Semiconductor–Metal Phase Transition and Emergent Charge Density Waves in 1*T*-ZrX_2 (X = Se, Te) at the Two-Dimensional Limit, *Nano Lett.* **2022**, 22 (1), 476-484, https://doi.org/10.1021/acs.nanolett.1c04372.

[S3] Wu, X.; Qiao, J.; Liu, L.; Shao, Y.; Liu, Z.; Li, L.; Zhu, Z.; Wang, C.; Hu, Z.; Ji, W.; Wang, Y.; & Gao, H.; Shallowing interfacial carrier trap in transition metal dichalcogenide heterostructures with interlayer hybridization, *Nano Res.* **2021**, 14, 1390–1396, https://doi.org/10.1007/s12274-020-3188-8.

[S4] Das, T.; Yang, E.; Seo, J. E.; Kim, J. H.; Park, E.; Kim, M.; Seo, D.; Kwak, J. Y.; and Chang, J.; Doping-Free All $PtSe_2$ Transistor via Thickness-Modulated Phase Transition, *ACS Appl. Mater. Interfaces* **2021**, 13, 1861–1871, https://doi.org/10.1021/acsami.0c17810.

[S5] Tran, T.; Bray, K.; Ford, M. J.; Toth, M.; & Aharonovich, I.; Quantum emission from hexagonal boron nitride monolayers, *Nature Nanotech* **2016**, 11, 37–41, https://doi.org/10.1038/nnano.2015.242
